# Andrographolide Sulfonates and Xiyanping: A Review of Chemical Composition, Pharmacological Activities, Clinical Applications, and Adverse Reactions

**DOI:** 10.3390/ph18020183

**Published:** 2025-01-29

**Authors:** Zihong Li, Lihao Yao, Zhenjie Liu, Liuping Wang, Huini Ruan, Yuanle Shen, Peng Zhang, Kaitong Li, Honglan Wang, Lili Fan, Liangxing Tu, Jianfang Feng

**Affiliations:** 1 School of Pharmacy, Guangxi University of Chinese Medicine, Nanning 530200, China; 2 Guangxi Engineering Technology Research Center of Advantage Chinese Patent Drug and Ethnic Drug Development, Nanning 530020, China; 3 National Pharmaceutical Engineering Center for Solid Preparation in Chinese Herbal Medicine, Jiangxi University of Chinese Medicine, Nanchang 330006, China

**Keywords:** andrographolide sulfonate, chemical composition, pharmacological activity, clinical application, adverse reaction, anti-inflammatory, antiviral activities

## Abstract

*Andrographis paniculata* is a plant of the Acanthaceae family and its primary bioactive constituent, andrographolide, exhibits a broad spectrum of pharmacological activities and notable clinical efficacy. However, its poor solubility and limited bioavailability pose significant challenges for therapeutic applications. To overcome these limitations, researchers have synthesized andrographolide sulfonates by reacting andrographolide with ethanol and sulfuric acid. This sulfonated derivative significantly enhances water solubility and bioavailability while retaining key pharmacological properties such as anti-inflammatory and antiviral activities. As a representative formulation, Xiyanping injection has been widely employed in the treatment of respiratory infections, pneumonia, and related conditions, playing a critical role during the COVID-19 pandemic. Despite its widespread application, there has yet to be a comprehensive review of its chemical composition and pharmacological mechanisms. Additionally, the safety of Xiyanping injection remains a topic of some debate. This review systematically examines the chemical composition, pharmacological activities, clinical applications, and adverse reactions of andrographolide sulfonates and their formulation in Xiyanping injection to provide a scientific basis for further research and applications, while also offering valuable insights for the development of similar sulfonated drugs.

## 1. Introduction

**Figure 1:**
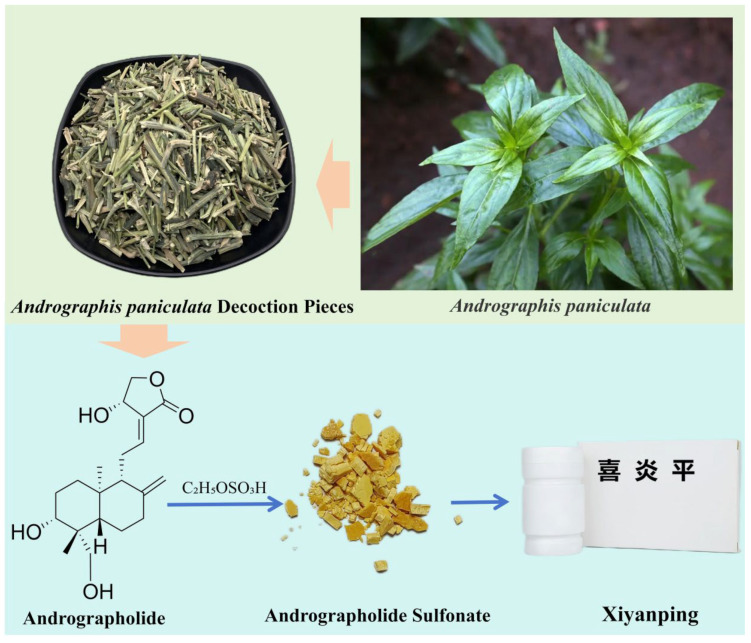
*Andrographis paniculata,* andrographolide, andrographolide sulfonate and Xiyanping injection (喜炎平).

*Andrographis paniculata* (Burm. f.) Wall. ex Nees in Wallich, an annual herbaceous plant of the Acanthaceae family, is native to India and is now widely distributed across tropical and subtropical regions of Asia. It thrives in warm, humid, and sunny environments. Renowned for its high medicinal value, *Andrographis paniculata* is used in traditional Indian medicine to treat conditions such as cancer, colds, and jaundice [1,2]. In Chinese traditional medicine, it is known for its heat-clearing and detoxifying properties, as well as its ability to reduce swelling and alleviate pain. It is commonly employed for treating ailments such as acute bacillary dysentery, enteritis, colds with fever, influenza, meningitis, pulmonary abscesses, and even venomous snake bites when used externally [3]. Modern medical research has identified andrographolide as the primary active compound in *Andrographis paniculata* [1,4,5,6]. This diterpenoid lactone demonstrates remarkable pharmacological activities, including anti-inflammatory, anticancer, and antiviral effects [7]. Due to its significant therapeutic potential, andrographolide has been developed into numerous pharmaceutical products, with 29 formulations currently approved in mainland China alone (https://db.yaozh.com/ accessed on 13 November 2024). However, andrographolide’s poor solubility, low intestinal permeability, susceptibility to P-glycoprotein efflux, and limited gastrointestinal stability result in exceptionally low oral bioavailability [8]; studies indicate that its bioavailability does not exceed three percent when administered orally in conventional dosage forms [9,10,11]. Furthermore, andrographolide is notoriously bitter, earning Andrographis paniculata the title of “King of Bitterness” in China [12], which significantly reduces patient compliance for its oral administration. To address these limitations, researchers have synthesized a series of sulfonated derivatives by reacting andrographolide with ethanol and sulfuric acid. These derivatives not only greatly enhance andrographolide’s water solubility but also preserve its pharmacological efficacy [13,14,15]. Andrographolide sulfonates are yellow, prone to clumping, and hygroscopic, requiring stringent storage conditions (Figure 1).

Xiyanping injection (XYP), developed by Jiangxi Qingfeng Pharmaceutical Co., Ltd. (Ganzhou, China) in China, is a pharmaceutical formulation composed solely of andrographolide sulfonates and water for injection, without additional excipients (https://db.yaozh.com accessed on 13 November 2024). Over the past two decades, XYP has been widely used in China to treat viral infections, acute respiratory infections, viral and bacterial pneumonia, and influenza. It also demonstrates synergistic effects when combined with antibiotics and has played a crucial role during the COVID-19 pandemic [16,17,18]. As a mixture, andrographolide sulfonates have garnered attention for their chemical composition, pharmacological activities, and the safety profile of their injectable formulations. Additionally, other pharmaceutical companies are exploring the development of inhalable and oral preparations of andrographolide sulfonates for both human and veterinary use [19]. Despite these advances, no comprehensive review has yet summarized the chemical, pharmacological, and clinical aspects of andrographolide sulfonates and their formulations. This article aims to systematically review the chemical composition, pharmacological activities, clinical applications, and adverse reactions of andrographolide sulfonates and XYP, providing a valuable reference for future research and applications.

## 2. Method of Study

A comprehensive search for both Chinese and English literature was conducted using the keywords andrographolide sulfonate, andrographolide total ester sulfonate, and XYP across multiple scientific databases, including Web of Science, Science Direct, Google Scholar, Cochrane Library, Springer, ClinicalTrials, PubMed, CNKI, and Wanfang. Inclusion criteria were as follows: studies that fully investigated the chemical composition, pharmacological activities, clinical applications, or adverse reactions of andrographolide sulfonate in China and other countries were included, regardless of the publication language. Additionally, authoritative sources such as the Chinese Pharmacopoeia and Yaozhi Database were utilized to gather further information. The collected data were meticulously categorized into different groups. The data collection process was finalized in September 2024. Chemical structures were drawn using ChemDraw 19. 0 software, while illustrations for the article were created using BioRender and Origin 2021.

## 3. Chemical Composition

**Figure 2:**
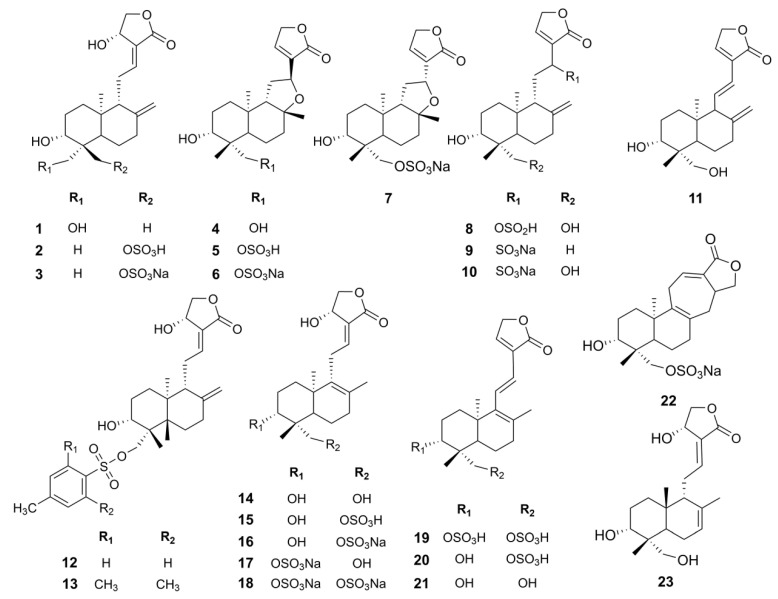
Chemical structure of andrographolide sulfonate.

**Figure 3:**
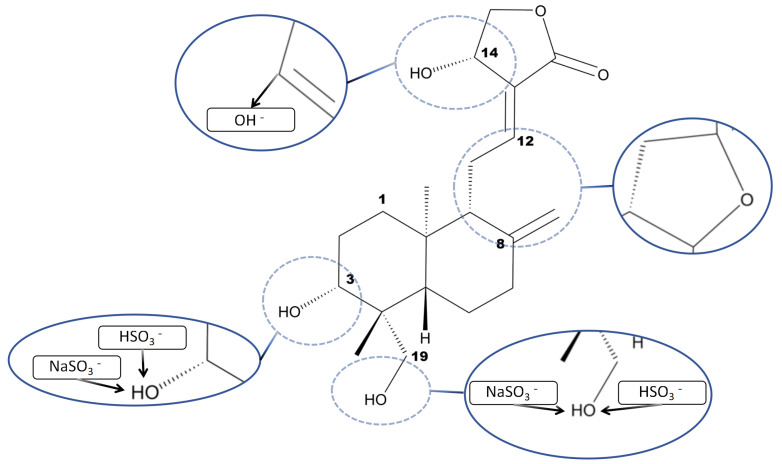
The chemical reaction process of the formation of andrographolide sulfate from andrographolide.

Andrographolide sulfonates are sulfonated derivatives of andrographolide. So far, it has been found to include 23 chemical compounds (Table 1, Figure 2). All these compounds share a diterpenoid lactone core structure. Apart from the parent compound andrographolide (**1**), the other 22 components exhibit structural and physicochemical similarities. Most of these compounds contain highly polar sulfonic acid groups, which significantly enhance the hydrophilicity of the diterpenoid lactone molecules. These polar groups form hydrogen bonds with water molecules, improving solubility. Additionally, sulfonation reduces hydrophobic interactions between molecules, facilitating better dispersion in aqueous media [20,21]. A structural analysis of the 22 sulfonated derivatives indicates that the C14-OH group of andrographolide is unstable under reaction conditions, readily undergoing dehydration to form conjugated double bonds and C8-O-C12 epoxide structures. This process increases the number of chiral centers, leading to the generation of various stereoisomers. Furthermore, the sulfonation reactions at C3-OH and C19-OH are not highly selective, resulting in mixtures of mono- and di-sulfonated derivatives within the same structural framework. These factors contribute to the complex chemical composition of andrographolide sulfonates [22] (Figure 3).

It is worth noting that not all components are sufficiently representative. Some are present in trace amounts or have unclear pharmacological activity, leading to their limited mention in studies. The critical components used for quality control in XYP can serve as important markers for andrographolide sulfonates. The original standard for XYP relied on ultraviolet spectrophotometry to quantify 17-hydro-9-dehydroandrographolide 19-sodium sulfate (**16**), also known as andrographolide sulfonate E, for quality control. Yang et al. [23] demonstrated that 17-hydro-9-dehydroandrographolide 19-sodium sulfate (**16**) can exist as a crystalline form with a stable three-dimensional supramolecular structure. It exhibits sufficient physicochemical stability, notable anti-inflammatory activity, and metabolic stability, and it is the most abundant compound in andrographolide sulfonates, reaching concentrations of up to 0. 8 mg/mL in XYP. However, ultraviolet spectrophotometry is prone to interference and lacks the accuracy and reproducibility of HPLC. Zhan et al. [24], using HPLC, identified four key components in XYP under specific chromatographic conditions: 17-hydro-9-dehydroandrographolide (**14**), 17-hydro-9-dehydroandrographolide 19-sodium sulfate (**16**), 17-hydro-9-dehydroandrographolide 3-sodium sulfate (**17**), and 17-hydro-9-dehydroandrographolide 3, 19-disodium sulfate (**18**). These compounds exhibited excellent separation, significant peak areas, good reproducibility, and notable antibacterial activity. Chong et al. [25] reported that in blood samples, 17-hydro-9-dehydroandrographolide (**14**) and 17-hydro-9-dehydroandrographolide 19-sodium sulfate (**16**) demonstrated high qualitative and quantitative accuracy without effects.

**Table 1:** Chemical composition of andrographolide sulfonate.

No.	Name	Chemical Formula	Reference
1	Andrographolide	C_20_H_30_O_5_	[26]
2	Andrographolide 19-sulfate	C_20_H_30_SO_8_	[26]
3	Sodium andrographolide 19-sulfate	C_20_H_29_SO_8_Na	[26]
4	Isoandrographolide	C_20_H_30_O_5_	[27]
5	8-epi-isoandrographolide-19-yl sulfate	C_20_H_30_SO_8_	[28]
6	Sodium 8-epi-isoandrographolide-19-yl sulfate	C_20_H_30_SO_8_Na	[28]
7	3-Hydroxy-8(R), 12(S)-8(12)-epoxy-13-labden-15, 16-olide-19-sulfate	C_20_H_30_SO_8_Na	[22]
8	Andrographolide 12-sulfate	C_20_H_30_SO_7_	[26]
9	Sodium andrographolide 12-sulfate	C_20_H_29_SO_6_Na	[26]
10	Andrographolide sodium bisulfate	C_20_H_29_SO_7_Na	[29]
11	14-Deoxy-11, 12-dehydroandrographolide	C_20_H_28_O_4_	[26]
12	Andrographolide 18-p-toluenesulfonate	C_27_H_36_SO_7_	[26]
13	Andrographolide 2, 4, 6-trimethylbenzenesulfonate	C_29_H_40_SO_7_	[26]
14	17-hydro-9-dehydroandrographolide	C_20_H_30_O_5_	[30]
15	17-Hydro-9-dehydroandrographolide 19-sulfate	C_20_H_30_SO_8_	[26]
16	17-hydro-9-dehydroandrographolide 19-sodium sulfate	C_20_H_29_SO_8_Na	[26]
17	17-hydro-9-dehydroandrographolide 3-sodium sulfate	C_20_H_29_SO_8_Na	[26]
18	17-hydro-9-dehydroandrographolide 3, 19-disodium sulfate	C_20_H_28_S_2_O_11_Na_2_	[26]
19	8, 11, 13-Labdatrien-15, 16-olide-3, 19-disulfate	C_20_H_28_S_2_O_10_	[22]
20	3-Hydroxy-8, 11, 13-labdatrien-15, 16-olide-19-sulfate	C_20_H_28_SO_7_	[22]
21	3, 19-Dihydroxy-8, 11, 13-labdatrien-15, 16-olide	C_20_H_28_O_4_	[22]
22	sodium 17-hydro-9-dehydro-14, 17-cyclo-andrographolide-19-yl sulfate	C_20_H_27_SO_7_Na	[28]
23	10β-8, 17-dihydro7, 8-dehydroandrographolide	C_20_H_30_O_5_	[31]

## 4. Pharmacological Activity

**Figure 4:**
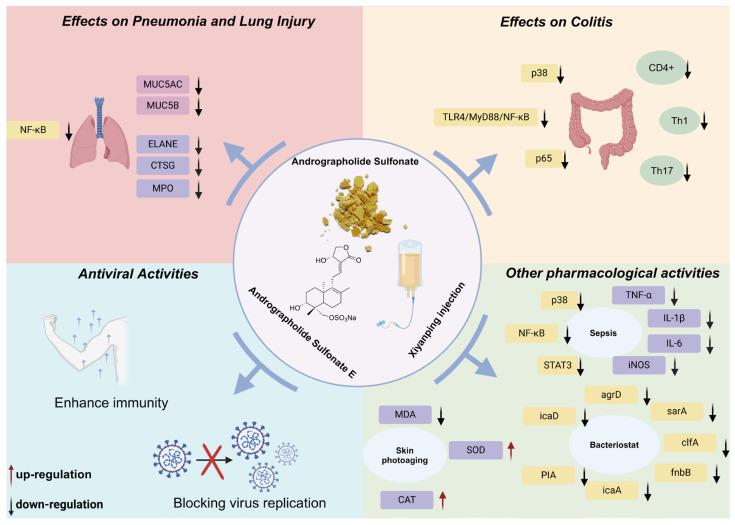
The pharmacological activities of andrographolide sulphonate and Xiyanping.

Andrographolide sulfonates exert their pharmacological effects primarily through two pathways: (1) undergoing metabolic conversion to regenerate andrographolide, thereby exerting the biological activities associated with the parent compound; and (2) exhibiting unique pharmacological activities distinct to the sulfonated derivatives. While andrographolide sulfonates retain many pharmacological properties of andrographolide due to the shared parent structure, their mechanisms and pathways of action are not entirely identical. This section focuses on the second pathway, summarizing recent studies on the pharmacological activities of andrographolide sulfonates. The pharmacological activities of andrographolide sulphonate and Xiyanping are shown in Figure 4.

### 4.1. Effects on Pneumonia and Lung Injury

Pneumonia is an inflammatory condition of the lungs caused by bacterial, viral, or fungal infections. Pathogens invade the lungs, leading to alveolar inflammation, fluid accumulation, and symptoms such as coughing, fever, and respiratory distress. Severe pneumonia can result in lung injury, which involves structural damage to lung tissue due to various etiologies [32].

Cui et al. [33] demonstrated that the intraperitoneal administration of andrographolide sulfonates effectively ameliorated airway inflammation and histopathological changes in a mouse model of pneumonia. The treatment also reduced pro-inflammatory cytokine levels in bronchoalveolar lavage fluid (BALF) and serum, while suppressing the expression of mucins MUC5AC and MUC5B in lung tissue. These effects were attributed to the inhibition of the NF-κB signaling pathway. Similarly, andrographolide sulfonates modulated NF-κB to alleviate alveolar coagulation, fibrinolysis inhibition [34], and acute lung injury [35], mirroring mechanisms observed with andrographolide in treating pneumonia [36,37]. Proteomic analyses further revealed that andrographolide sulfonates mitigated LPS-induced acute lung injury in mice by inhibiting neutrophil-released proteases, such as elastase (ELANE), cathepsin G (CTSG), and myeloperoxidase (MPO) [38].

Andrographolide sulfonates also exhibit synergistic effects with other drugs in pneumonia treatment [39]. For example, Zhang et al. [40] reported that combining andrographolide sulfonates with imipenem significantly improved survival rates and reduced MCP-5 levels in mice infected with *Klebsiella pneumoniae*. Similarly, Gu et al. [41] found that combining andrographolide sulfonates with azithromycin enhanced therapeutic outcomes in pneumonia caused by K. pneumoniae. Notably, the combination reduced the hepatic accumulation of azithromycin, suggesting that andrographolide sulfonates may promote azithromycin metabolism, potentially protecting the liver and mitigating drug resistance. However, another study highlighted that andrographolide sulfonates might inhibit CYP3A4 activity, slowing the metabolism of drugs such as lopinavir and ritonavir in rats, which could pose risks [42]. Therefore, the careful evaluation of drug interactions is essential when using andrographolide sulfonates.

### 4.2. Effects on Colitis

Colitis, an inflammatory bowel disease (IBD), is characterized by symptoms such as abdominal pain, diarrhea, and bloody stools. Ulcerative colitis, the most common form of colitis, involves factors such as immune dysregulation, genetic predisposition, and gut microbiota imbalances [43].

**Figure 5:**
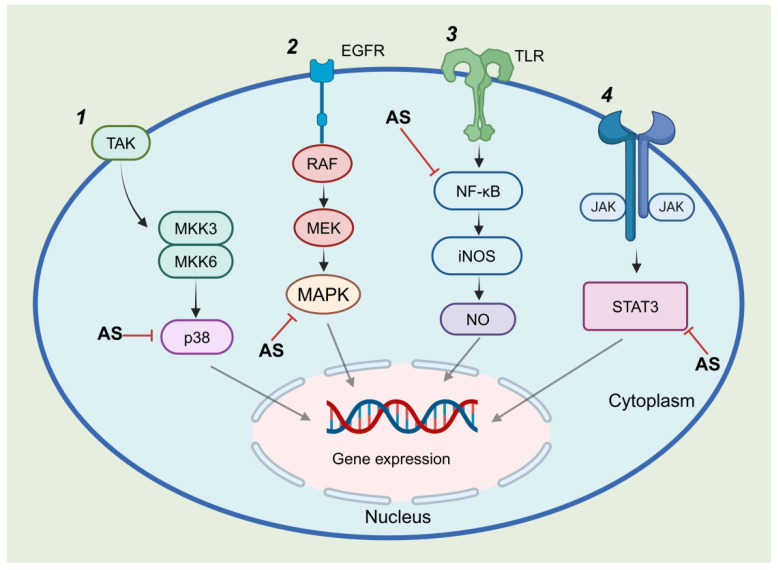
Anti-inflammatory mechanisms of andrographolide sulfonates. 1: Andrographolide sulfonates alleviate inflammation by inhibiting p38 phosphorylation. 2: Andrographolide sulfonates alleviate inflammation by inhibiting MAPK phosphorylation. 3: Andrographolide sulfonates reduce inflammation by inhibiting NF-κB phosphorylation. 4: Andrographolide sulfonates alleviate inflammation through negative regulation of STAT3 activation.

Unlike the relatively singular mechanisms observed in pneumonia treatment, andrographolide sulfonates exhibit diverse pharmacological mechanisms in colitis therapy. Guan et al. [44] showed that andrographolide sulfonates reduced colitis in DSS-induced mouse models by inhibiting pro-inflammatory macrophage polarization within the liver–gut axis, suppressing YAP-mediated colitis responses and downregulating the TLR4/MyD88/NF-κB signaling pathway to reduce inflammatory cytokine release. Liu [45] further explored the mechanisms in TNBS-induced colitis models, revealing that andrographolide sulfonates suppressed excessive Th1/Th17 cell responses and inhibited p38 and NF-κB signaling pathways. This demonstrates the compound’s ability to modulate multiple inflammatory responses within the immune system to alleviate colitis. Gao et al. [46] reinforced these findings, noting that andrographolide sulfonates significantly reduced inflammatory cytokine levels in colon tissue, inhibited CD4+ T cell and macrophage infiltration, and suppressed the activation of the p38 and p65 signaling pathways. In summary, andrographolide sulfonates exert potent anti-inflammatory effects in colitis by targeting multiple pathways, including YAP, TLR4/MyD88/NF-κB, and p38 signaling. These mechanisms differ from andrographolide’s actions, which primarily involve activating Nrf2/HO-1-mediated antioxidant responses or repairing DNA damage in colonic epithelial cells to mitigate oxidative stress and inflammation [47]. Figure 5 summarizes the anti-inflammatory mechanisms of andrographolide sulfonates [34,35,48].

### 4.3. Antiviral Activities

Viral infections are a significant cause of various diseases due to their high transmissibility. Viruses invade host cells, replicating and forming new viral particles that destroy host cells, triggering inflammatory responses and tissue damage [49]. Adenoviruses (AdV), non-enveloped double-stranded DNA viruses, are common opportunistic pathogens that can infect the respiratory tract, gastrointestinal tract, eyes, bladder, and liver, leading to widespread epidemics [50].

Ping et al. [14] found that andrographolide sulfonates significantly inhibit the proliferation of adenovirus-3 (AdV3) in Hep-2 cells. The proposed mechanism suggests that andrographolide sulfonates preferent permeability and reduced related metabolic products, such as anthranilic acid and D-lactic acid. Hand, foot, and mouth disease (HFMD) is an acute contagious illness caused by various enteroviruses, primarily affecting infants and young children. It is characterized by fever, rashes, and ulcers on the hands, feet, and inside the mouth. Enterovirus 71 (EV71) is identified as the main causative agent of HFMD [51]. A study by Li et al. [52] revealed that andrographolide sulfonate does not inhibit EV71 viral replication in vitro. However, clinical data reports demonstrate that andrographolide sulfonate has shown significant therapeutic effects in treating HFMD [53,54]. Consequently, the researchers conducted animal experiments to explore the mechanisms underlying its therapeutic action. Their findings suggest that andrographolide sulfonate primarily enhances immune regulation by modulating neutrophils and T-lymphocytes, thereby boosting the host’s immunity and indirectly protecting mice from EV71 infection. Clinical trial outcomes have further supported this conclusion [55]. Additionally, Li et al. [56], utilizing network pharmacology, molecular docking, and in vitro validation, identified that 17-hydro-9-dehydroandrographolide-19-sodium sulfate (**16**) is a key active component of andrographolide sulfonate against the COVID-19 virus. Their study pinpointed IL-6, TNF, IL-1β, CXCL8, and p-STAT3 as core molecular targets responsible for its antiviral activity. Together, these findings broaden our understanding of andrographolide sulfonate’s potential therapeutic mechanisms in combating viral diseases such as HFMD and COVID-19.

### 4.4. Other Pharmacological Activities

In addition to the pharmacological activities mentioned above, andrographolide sulfonates exhibit a variety of other therapeutic effects through distinct mechanisms. Xiao et al. [28] demonstrated that sodium 8-epi-isoandrographolide-19-yl sulfate (**6**), sodium 8, 12-epi-isoandrographolide-19-yl sulfate (**7**), 17-hydro-9-dehydroandrographolide (**14**), and 17-hydro-9-dehydroandrographolide 19-sodium sulfate (**16**) exhibit significant anti-angiogenic activity, highlighting their potential for development as anticancer agents. Sepsis, a systemic inflammatory response syndrome induced by infection, is typically caused by bacterial, viral, or fungal pathogens. It is characterized by immune dysregulation, leading to systemic inflammation and multi-organ dysfunction. Severe cases may progress to septic shock, marked by persistent hypotension and acute organ failure. Despite advances in medicine, sepsis remains a major global health challenge with a high mortality rate [57]. Guo et al. [48] found that andrographolide significantly reduced serum levels of pro-inflammatory cytokines TNF-α and IL-1β in septic mice, decreased transaminase levels, and alleviated liver and lung injury, thereby improving survival rates. Furthermore, andrographolide sulfonates demonstrated potent anti-inflammatory and organ-protective effects by inhibiting TNF-α, IL-1β, IL-6, and inducible nitric oxide synthase (iNOS) expression in damaged liver tissue. These effects are primarily mediated through the suppression of the p38 MAPK, STAT3, and NF-κB signaling pathways. Methicillin-resistant *Staphylococcus aureus* (MRSA) is a highly virulent pathogen responsible for serious infections both in and outside healthcare settings. Its resistance to multiple antibiotics, including penicillin, poses a significant therapeutic challenge [58]. Zhang et al. [59] reported that andrographolide sulfonates exhibit strong inhibitory activity against MRSA. The antibacterial mechanism involves downregulating quorum-sensing regulatory genes (agrD and sarA), microbial surface component recognition and adhesion-related genes (clfA and fnbB), and biofilm-associated genes (icaA, icaD, and PIA). These actions suppress the biosynthesis of five key biofilm-related metabolites, including anthranilic acid and D-lactic acid, disrupting biofilm formation and altering its permeability. Although the anti-inflammatory properties of andrographolide sulfonates are well documented, their protective effects against ultraviolet (UV) radiation-induced oxidative stress and skin inflammation have only recently been explored. Zhan et al. [60] investigated the effects of andrographolide sodium bisulfate (**10**) on UV-induced photoaging in mice. Physiological and histological analyses revealed that the intraperitoneal administration of andrographolide sodium bisulfate significantly reduced skin thickening, loss of elasticity, wrinkle formation, and dehydration caused by UV exposure. Additionally, it decreased malondialdehyde (MDA) levels, increased superoxide dismutase (SOD) and catalase (CAT) activity, and downregulated pro-inflammatory cytokines IL-1β, IL-6, IL-10, and TNF-α. 5-Fluorouracil (5-FU) is a commonly used chemotherapeutic agent, but resistance to this drug often limits its effectiveness [61]. Xu et al. [62] found that andrographolide sulfonates have potential synergistic effects in overcoming 5-FU resistance. In a mouse model bearing CT-26 tumor cells, 5-FU was shown to activate NLRP3 inflammasomes in myeloid-derived suppressor cells (MDSCs), contributing to resistance. Andrographolide sulfonates effectively inhibited NLRP3 activation, reversing drug resistance and enhancing the antitumor efficacy of 5-FU.

These studies highlight the diverse pharmacological activities of andrographolide sulfonates, providing a strong theoretical basis for their potential applications in medicine. However, the precise mechanisms of action and the roles of key active components within these complex mixtures remain to be fully elucidated. Further in-depth studies are required to optimize their therapeutic applications and clarify their mechanisms of action.

## 5. Clinical Application

### 5.1. Current Status of Clinical Applications

**Figure 6:**
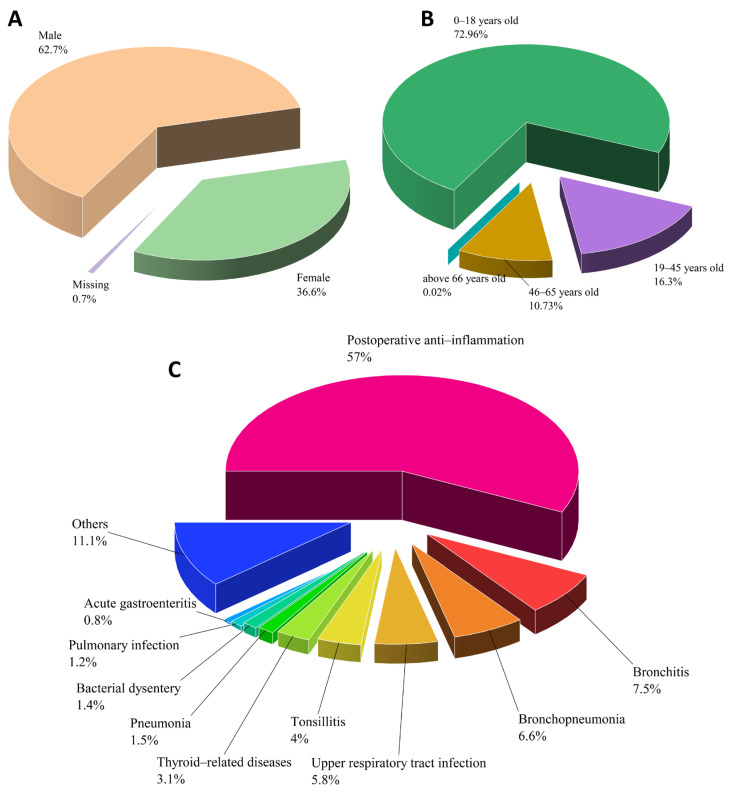
Clinical application of XYP; (**A**) Distribution of patient gender (4023 cases) [64]; (**B**) Distribution of patient age (4023 cases) [64]; (**C**) Distribution of patient medication purposes (848 cases) [65].

XYP, the only marketed preparation with andrographolide sulfonates as its main active ingredient, serves as a representative for the clinical research of these compounds. Clinically, XYP is primarily used to treat bronchitis, tonsillitis, pneumonia, and bacillary dysentery. Some studies also suggest its effectiveness in treating viral diseases such as hand, foot, and mouth disease (HFMD) and COVID-19, reflecting the clinical indications of andrographolide [4]. The injection is administered via intravenous or intramuscular routes, diluted with saline or 5% glucose solution. Intramuscular doses for adults range from 50–100 mg, two to three times daily, with pediatric doses adjusted accordingly. Intravenous administration ranges from 250 to 500 mg per day for adults, while children receive 5–10 mg/kg daily. Other less common administration methods include nebulization and micro-pump infusion for pneumonia treatment [63]. According to literature reports [64,65], the distribution of gender, age, and medication purposes for clinical cases of XYP is shown in Figure 6. It is worth noting that in all cases, XYP was used in combination with other medications. This is primarily because XYP is an exclusive hospital-use formulation, often serving as a crucial component in systemic treatment regimens rather than being the sole therapeutic agent. The drugs most frequently combined with XYP, listed in descending order of frequency, include ribavirin injection, cephalosporins, and ambroxol injection [66]. This combination approach poses challenges in managing the adverse reactions associated with XYP.

### 5.2. Clinical Trial Research

Based on the search results from ClinicalTrials (https://clinicaltrials.gov/ accessed on 1 November 2024) and TrialSearch (https://trialsearch.who.int/ accessed on 1 November 2024), we selected a subset of representative clinical trials with sound study designs for summarization. The results are shown in Table 2. It is also worth noting that some high-quality clinical studies have not yet published their results or academic papers based on the trials, so the findings remain unavailable.

#### 5.2.1. Treatment of Pneumonia

XYP has shown significant efficacy in treating pneumonia of various etiologies. For instance, human bocavirus (HBoV), discovered in 2005, is a common pathogen causing pneumonia in immunocompromised children, presenting with symptoms such as fever, respiratory distress, vomiting, and lethargy [67]. A randomized controlled trial by Wang et al. at Jiangsu Provincial Hospital of Traditional Chinese Medicine involved 59 children with HBoV pneumonia, divided into a control group receiving ambroxol oral solution and intravenous ribavirin and a treatment group receiving Qingfei oral liquid combined with intravenous XYP. The treatment group demonstrated a significantly higher cure rate (38.71%) compared to the control group (17.86%), along with lower symptom scores, indicating the efficacy of the combination therapy [68]. Another study focused on *Mycoplasma pneumoniae* pneumonia (MPP), a primary cause of pediatric pneumonia characterized by severe coughing. This condition predominantly affects school-age children but has shown a trend toward younger age groups, including infants and neonates [69]. In a randomized trial involving 72 children with MPP, the control group was treated with azithromycin alone, while the treatment group received azithromycin combined with intravenous XYP. After 7–11 days, both groups showed improvement, but the treatment group demonstrated superior recovery rates and reduced symptom duration. Additionally, serum levels of IL-2 and IFN-γ increas ed significantly, while IL-4 and IL-10 levels decreased in both groups. These results highlight the efficacy of combining XYP with azithromycin in treating MPP [70].

#### 5.2.2. Treatment of Bronchitis

Bronchitis, a common respiratory condition, is characterized by inflammation of the trachea, bronchi, and surrounding tissues, often caused by microbial infections or irritants [71]. It presents with symptoms such as coughing, sputum production, and wheezing [72]. A multicenter, randomized, parallel-controlled study involving 78 children aged 1–6 with acute bronchitis demonstrated a 100% overall efficacy rate in the group receiving intramuscular XYP combined with conventional therapy, compared to 73% in the control group after three days of treatment [73]. Another double-blind, randomized controlled trial involving 148 adults with acute bronchitis found that XYP significantly reduced the duration of symptoms such as coughing, chills, and fever [74]. Furthermore, combining piperacillin–tazobactam with XYP showed superior improvements in clinical indicators such as fever reduction, cough relief, and resolution of lung rales compared to piperacillin–tazobactam alone [75].

#### 5.2.3. Treatment of Hand, Foot, and Mouth Disease

HFMD is a globally prevalent acute viral illness caused by enteroviruses, primarily affecting children under five years of age. Its clinical features include fevers, rashes, and ulcers on the hands, feet, and oral mucosa [51,76]. A prospective randomized controlled trial involving 451 children with severe HFMD found that the group receiving XYP combined with standard therapy had a higher overall efficacy rate (43.6%) compared to the standard therapy group (29.5%). Symptom relief rates for cough, lethargy, irritability, and fatigue were also significantly higher in the combination group [77]. Another randomized trial evaluating XYP in mild HFMD cases revealed shorter times to fever resolution and rash/ulcer disappearance in the XYP group compared to the control group receiving vitamins and ibuprofen [78].

#### 5.2.4. Other Clinical Applications

COVID-19, the most widespread and impactful viral disease of the 21st century, causes respiratory symptoms ranging from mild to severe pneumonia [79]. A multicenter, open-label, randomized controlled trial by Zhang et al. involving 130 mild-to-moderate COVID-19 patients demonstrated that XYP combined with standard therapy significantly reduced progression to severe disease and shortened the time to symptom resolution and viral clearance [80]. XYP has also shown promise in treating tonsillitis [81] and acute gastroenteritis [82] when combined with antibiotics.

**Table 2:** Clinical trials of XYP.

No.	Disease	Inclusion Criteria and Number of Patients	Method	Control Drug	Combined Drug	Efficacy	Reference
1	COVID-19	130 adults aged 18 and above with mild to moderate COVID-19	multicenter, prospective, open-label, and randomized controlled trial	Conventional therapy: Includes oxygen supplementation, antiviral drugs, antibiotics, immune modulators, etc.	Conventional therapy	XYP significantly shortened the time it took for cough relief, fever reduction, and viral clearance.	[80]
2	Hand, Foot, and Mouth Disease	230 children aged 1–13 years with severe hand, foot, and mouth disease	Randomized Controlled Trial	Conventional therapy: Mannitol, methylprednisolone, hydrocortisone, dexamethasone, and intravenous immunoglobulin, etc.	Conventional therapy	Adding andrographolide sulfonate to conventional treatment can reduce the occurrence of major complications in children with severe hand, foot, and mouth disease, shorten the duration of fevers, and accelerate the healing time of typical skin or oral mucosal lesions.	[53]
3	Hand, Foot, and Mouth Disease	451 children aged 1–13 years with severe hand, foot, and mouth disease	Prospective, Randomized, Controlled Trial	Conventional therapy (according to the 2010 guidelines for the diagnosis and treatment of hand, foot, and mouth disease issued by the National Health and Family Planning Commission of China)	Conventional therapy	The efficacy of XYP in combination with conventional treatment is superior to conventional treatment alone.	[77]
4	Hand, Foot, and Mouth Disease	329 children aged 1–14 years with mild hand, foot, and mouth disease	Randomized controlled trial	Vitamin B and C, physical cooling, and combined use of ibuprofen	Conventional therapy	XYP can shorten the onset time of antipyretic effects, as well as the regression time of hand–foot rashes and oral ulcers. The combination of XYP and the control drug further shortens the regression time of rashes and ulcers.	[78]
5	Acute Gastroenteritis	200 college students diagnosed with acute gastroenteritis	Randomized controlled trial	Racemic scopolamine hydrochloride	Jinniu abdominal pain tablets	XYP combined with Jinniu abdominal pain tablets in the treatment of acute gastroenteritis significantly reduces serum CRP levels, improving various treatment indicators and yielding good therapeutic effects. The combination group also has a higher treatment efficacy rate than the control group.	[82]
6	Acute Tonsillitis	458 patients of all ages with acute tonsillitis	Multicenter, randomized, single-blind, placebo, parallel-controlled trial	Clindamycin, azithromycin, and other antibiotics combined with placebo	Clindamycin, azithromycin, and other antibiotics	XYP can shorten the disease remission time in patients with acute tonsillitis, improve clinical cure rates, and accelerate the alleviation of various symptoms, with clinically reliable safety and efficacy.	[81]
7	Acute Bronchitis in Children	78 children aged 1–6 years with acute bronchitis	Multicenter, randomized, parallel-controlled trial	Conventional therapy: Cough suppressants, expectorants, antipyretics, bronchodilators, antihistamines, anti-diarrheal drugs, and oral traditional Chinese medicine without andrographolide	Conventional therapy	The combination of conventional treatment and XYP via intramuscular injection in the treatment of pediatric acute bronchitis is more effective than conventional treatment alone, without increasing adverse reactions.	[73]
8	Lower Respiratory Tract Infection	108 patients aged 47–70 years with lower respiratory tract infection	Multicenter, randomized, parallel-controlled trial	Conventional therapy: Cephalosporin antibiotics combined with traditional Chinese and Western medicine for antipyretic, lung-clearing, and cough-suppressing treatment	Conventional therapy	The combination of conventional treatment and XYP for lower respiratory tract infections shows clear clinical efficacy and is superior to conventional treatment alone.	[83]
9	HBoV Pneumonia	59 children aged 1–5 years with mild to moderate HBoV pneumonia	Randomized controlled trial	Oral ambroxol solution combined with intravenous ribavirin injection	Qingfei oral solution	Qingfei oral solution combined with XYP in the treatment of pediatric bocavirus pneumonia is effective and superior to treatment with oral ambroxol solution combined with intravenous ribavirin injection.	[68]
10	Pediatric Mycoplasma Pneumonia	72 patients aged 1–16 years with mycoplasma pneumonia	Randomized controlled trial	Azithromycin	Azithromycin	XYP combined with azithromycin significantly improves the clinical symptoms in children with mycoplasma pneumonia associated with wind-heat obstructing the lung and phlegm-heat obstructing the lung syndromes in traditional Chinese medicine, with a better treatment effect in the combination group compared to the azithromycin monotherapy group.	[70]

XYP is widely used in the treatment of various infectious diseases. As a “herbal antibiotic”, it offers a distinct advantage over traditional antibiotics by reducing the risk of resistance. Its clinical indications and administration methods, such as nebulization, continue to expand with ongoing research. However, many existing studies suffer from limitations, including being of low quality, single-source funding, and design flaws (on the Jadad seven-point scale, most studies in the literature scored essentially less than three points), which may undermine the reliability of their conclusions and clinical recommendations. Thus, large-scale, high-quality clinical trials are urgently needed to further elucidate the therapeutic potential of andrographolide sulfonates.

## 6. Adverse Reaction

Safety remains a critical concern for traditional Chinese medicine (TCM) injections. XYP emerged during a unique historical period when approximately 1400 TCM injections were developed, most of which were subsequently discontinued and failed to enter 21st-century clinical applications [84]. In 2017, XYP was briefly withdrawn due to quality issues in certain product batches. However, following rigorous testing, on-site inspections, and risk assessments conducted by Chinese regulatory authorities, its market approval was promptly reinstated [85].

Clinical data suggest that adverse reactions associated with XYP occur in the following order of frequency: damage to the skin and its appendages, anaphylactic shock, digestive system damage, and respiratory system injury. Treatment for these reactions typically involves dexamethasone, epinephrine, calcium gluconate, or dopamine, with oxygen supplementation and fluid expansion in severe cases. The cure rate for such adverse reactions is generally high [86]. Some researchers attribute these reactions to the presence of macromolecular components in XYP, which form the basis for hypersensitivity responses [87]. Specifically, the incomplete removal of hapten-like substances in the injection may result in their binding with plasma proteins to form antigen–antibody complexes. These complexes activate the complement system, producing anaphylatoxins that trigger the activation of mast cells, basophils, and platelets, releasing inflammatory mediators like histamine. This cascade leads to local edema and attracts neutrophils, which release proteolytic enzymes, collagenases, and elastases, further damaging vascular and surrounding tissue cells. The resulting cell lysis exacerbates the damage, potentially leading to severe allergic reactions [88,89,90].

The quality of injectable drugs can be significantly compromised by improper pH levels or compatibility issues with other medications, leading to the formation of insoluble particles. These particles may, in turn, cause pyrogenic or anaphylactic-like reactions when introduced into the body [86]. Experimental evidence has shown that when XYP is combined with cefazolin sodium injection, cefuroxime sodium injection, or cefuroxime sodium injection, the pH level increases. Conversely, when combined with sodium penicillin or vitamin B6 injections, the pH level decreases. Additionally, when combined with acyclovir or ganciclovir injections, there is a significant reduction in the content of its main sulfonated component, E. Notably, the researcher also observed that the choice of solvent does not affect the safety of XYP, but different solvents may influence its safety when used in combination with other drugs. In an experimental study, XYP diluted with 5% glucose or 0.9% sodium chloride maintained its stability. Over a 6 h period, the preparation’s properties, pH, insoluble particles, and the content of the active compound, andrographolide sulfonate E, remained unchanged. However, when XYP was combined with 15 other drugs, such as ambroxol hydrochloride injection, using either 5% glucose or 0.9% sodium chloride as diluents, there was a notable increase in the number of insoluble particles [91]. Therefore, experts strongly recommend that XYP should never be mixed with other injections in the same container [92]. Unlike modifications that target specific sites or functional groups of andrographolide, sulfonation introduces greater chemical complexity to the molecule. While this increases its pharmacological potential, it also raises its risk profile. Moreover, because andrographolide sulfonates are often administered intravenously, bypassing the intestinal barrier, the safety requirements for these compounds are particularly stringent. This highlights the necessity for extensive research into their safety.

Despite being administered primarily to children—an immunocompromised and physiologically vulnerable population—the incidence of adverse reactions to XYP remains relatively low. For example, a study conducted from September 2012 to October 2013 across several major hospitals in China reported only seven cases of adverse reactions out of 4023 patients, representing an incidence rate of 0.17% [64]. Nonetheless, due to the widespread use of XYP, a significant sample size of adverse reaction cases has been accumulated. Among 109 patients experiencing adverse reactions, the types and clinical manifestations of these reactions are summarized in Table 3 [86]. Most adverse reactions were mild, involving allergic skin damage, while severe cases of anaphylactic shock were rare. Pre-treatment allergy testing and stringent quality control can significantly reduce the likelihood of adverse reactions. To enhance the safety of TCM injections like XYP, it is essential to establish a standardized and comprehensive framework for re-evaluating non-clinical safety. This should include studies on toxicity, systemic and skin allergies, and vascular irritation. Such research must adhere strictly to Good Laboratory Practice (GLP) standards and relevant guidelines. Additionally, all original data and documentation should be meticulously recorded and preserved to ensure the reliability and authenticity of the research findings [93]. By implementing these measures, the safety and clinical applicability of andrographolide sulfonates can be further improved, thereby reducing the risks associated with their use.

## 7. Conclusions and Future Prospects

As a chemically modified natural product, andrographolide sulfonates have been widely utilized in China. This review examines their chemical composition, pharmacological activities, clinical applications, and adverse reactions, while exploring prospects for future research and development.

However, several issues require further investigation. First, XYP, the marketed formulation of andrographolide sulfonates, is primarily used in China and a few Southeast Asian countries, with limited recognition in Western markets like Europe and the United States. Second, XYP is rarely used as a standalone treatment; it is often combined with traditional Chinese medicine or chemical drugs, which may increase the risk of adverse reactions. Third, the therapeutic indications for andrographolide sulfonates remain narrow, with significant overlap with andrographolide, limiting their differentiation in clinical use. Lastly, clinical research on andrographolide sulfonates is limited, with small sample sizes, short observation periods, and a lack of robust large-scale, multicenter, randomized controlled trials or long-term follow-up studies, which restricts their broader adoption.

To address these challenges, the following solutions are proposed:

(1) Conduct international collaborative research to enhance our understanding of andrographolide sulfonates, establish quality standards, develop production protocols, and seek overseas approval;
(2) Perform large-scale safety assessments to clarify contraindications for combined medication use and implement strict clinical controls to prevent adverse reactions;
(3) Investigate new therapeutic indications and innovative applications, such as veterinary drugs or agrochemicals, while comparing efficacy with existing treatments;
(4) Initiate large-scale, multicenter, randomized controlled trials to validate safety, efficacy, and long-term risks.

Research on andrographolide sulfonates provides valuable insights for improving other poorly water-soluble drugs. Many existing drugs for infectious diseases, such as baicalin [94], artemisinin [95], and azithromycin [96], share the common limitation of poor water solubility. This characteristic poses challenges, particularly for treating systemic infections in which the drug must reach the infection site via the bloodstream. Poor water solubility also limits drug dissolution and absorption following oral administration, leading to reduced bioavailability [97,98]. Injectable formulations may face difficulties in finding safe and appropriate solvents. Additionally, water-soluble drugs are more readily distributed in aqueous compartments such as blood and interstitial fluid, while lipophilic drugs tend to accumulate in fatty tissues [99,100]. These factors collectively hinder the therapeutic efficacy of poorly water-soluble drugs for infectious diseases. Inspired by andrographolide sulfonates, chemical modifications, or other techniques to improve water solubility can significantly expand administration options. Depending on the context, formulations such as injections, oral tablets, or nebulizations could enhance therapeutic outcomes for previously underperforming drugs.

The market does not lie. XYP, renowned for its distinctive therapeutic efficacy and its unique position as a “natural antibiotic”, has gained widespread recognition in China’s healthcare sector. In 2023, XYP achieved annual sales exceeding 3.5 billion RMB (about USD 480 million) in China’s public medical institutions, ranking first among similar medications in terms of sales volume (Menet, https://www.menet.com.cn/ accessed on 4 January 2025). Furthermore, literature-based research indicates that Xiyanping injection demonstrates significant advantages in both efficacy and cost-effectiveness compared to conventional therapies for treating pediatric bronchitis [101]. With its strong clinical performance and robust market presence, XYP has established itself as a cornerstone of the pharmaceutical market in China.

In summary, andrographolide sulfonates are a class of pharmacological compounds that have demonstrated broad clinical applications and favorable therapeutic effects. Conducting comprehensive studies on the solubility, bioavailability, and therapeutic efficacy of all included compounds, alongside thorough safety evaluations, to identify the most effective andrographolide sulfonate monomer and develop it into a clinical formulation, may represent a promising research direction. Their development provides a template for advancing similar compounds. With appropriate advancements in medicinal chemistry, pharmacology, toxicology, and clinical research, andrographolide sulfonates have significant potential for broader applications, contributing to the advancement of human healthcare.

## Figures and Tables

**Table 3:** Percentage of adverse reactions and clinical manifestations of XYP [86].

Type of Adverse Reaction	Proportion (%)	Clinical Manifestation
Skin and Appendage Damage	50.57	Primarily manifested as facial flushing, accompanied by rashes, urticaria, and wheal-like erythema distributed on the face and neck. In some patients, the rash extends to the chest and limbs. Vaso-neurotic edema is observed, with pronounced eyelid and facial swelling, cold edema of the upper and lower lips, and mild swelling of the fingers and toes.
Anaphylactic Shock	21.59	Symptoms include nausea, vomiting, restlessness, cyanosis of the lips, bluish discoloration of the face, cold extremities, hypotension, and impaired consciousness.
Digestive System	11.36	Accompanied by diarrhea, watery stools, nausea, severe vomiting, and hyperactive bowel sounds.
Respiratory System	7.95	Coughing and shortness of breath.
Other Severe Anaphylactoid Reactions	3.98	Symptoms include dizziness, abdominal pain, fine facial sweating, dry mouth, temporary slowed cognitive response with cold sweating, forced limb convulsions, lethargy, pale complexion, cold and clammy extremities, and mild hypotension (not reaching shock levels).
Cardiovascular System	3.41	Tachycardia.
Allergic Asthma	0.57	Chest tightness, breathlessness, and audible significant wheezing sounds in both lungs.
Drug Fever	0.57	High fever with abnormally elevated body temperature

## List of Abbreviations

COVID-19	Coronavirus Disease 2019
MUC5AC	Mucin 5AC
MUC5B	Mucin 5B
NF-κB	Nuclear Factor Kappa B
MCP-5	Monocyte Chemoattractant Protein 5
CYP3A4	Cytochrome P450 3A4
p38	p38 Mitogen-Activated Protein Kinase (p38 MAPK)
p65	p65 Subunit of NF-κB
YAP	Yes-Associated Protein
TLR4	Toll-Like Receptor 4
MyD88	Myeloid Differentiation Primary Response 88
Nrf2/HO-1	Nuclear Factor Erythroid 2-Related Factor 2/Heme Oxygenase-1
TNF-α	Tumor Necrosis Factor Alpha
IL-1β	Interleukin-1 Beta
IL-6	Interleukin-6
IL-10	Interleukin-10
IFN-γ	Interferon Gamma
